# Using Real Electronic Health Records in Undergraduate Education: Roundtable Discussion

**DOI:** 10.2196/60789

**Published:** 2025-06-12

**Authors:** Fatima Nadeem, Jessica Azmy, Asieh Yousefnejad Shomali, Benjamin Diette, Lloyd J Gregory, Angela C Davies, Kurt C Wilson

**Affiliations:** 1 Faculty of Biology, Medicine and Health, University of Manchester, Oxford Road, Manchester, M139PL, United Kingdom, 44 01613066000; 2 Health Innovation Manchester, Manchester, United Kingdom

**Keywords:** electronic health records, clinical competence, medical records systems, health care education, interdisciplinary education, undergraduate, student, health care professional, medical education, clinical practice, patient records, digital health, health informatics

## Abstract

**Background:**

Simulated electronic health records (EHRs) are used in structured teaching for health care students. This partly addresses inconsistent student exposure to EHRs while on clinical placements. However, simulated records are poor replacements for the complexity of data encountered in real EHRs. While routinely collected health care data are often used for research, secondary use does not include education. We are exploring the perceptions, governance, and ethics required to support the use of real patient records within teaching.

**Objective:**

The aim of the study is to explore the perspectives of health care professionals regarding the use of real patient records to deliver interprofessional EHR education to undergraduate health care students.

**Methods:**

We held 90-minute group discussions with 10 health care professionals from nursing, pharmacy, medicine, and allied health disciplines. We used the GRIPP2 (Guidance for Reporting Involvement of Patients and the Public 2) checklist for reporting Patient and Public Involvement and Engagement to present our reflections.

**Results:**

There was consensus on the need to upskill health care students in the use of EHRs. Participants emphasized teaching general EHR competencies and transferable skills to overcome the diversity in EHR systems. They highlighted limitations in current teaching due to accessibility issues, disparities within clinical teaching, and curricular gaps on important topics such as clinical documentation and coding. Highlighted benefits of using real EHRs in teaching included learning from the complexities and inaccuracies of real patient data, grasping real-world time frames, and better appreciation of multidisciplinary interactions. Concerns included exposing individual clinicians to unfounded scrutiny and the potential consequences of incidental findings within EHRs. The ethical implications of overlooking perceived errors within EHRs versus the impracticality of acting on them were discussed. To mitigate concerns, it was suggested that data donors would provide informed consent ensuring they understand that they will not be recontacted should any such errors be found.

**Conclusions:**

Innovative solutions are needed to realign health care education with clinical practice in rapidly evolving digital environments. Real patient records are optimal for teaching students to handle complex and abundant real-world data. Data within EHRs represent a wealth of clinical knowledge encompassing professional and personal experiences spanning the lifetimes of patients and their caregivers. Drawing experiences and events from real EHRs will prepare health care students to anticipate, confront, and manage real patients in a variety of real-life scenarios. Our reflections highlight the processes and safeguards to consider when using real patient records to deliver EHR education to health care students. These detailed reflections from discussions with health care professionals provide the grounds for a robust framework, with appropriate governance and consent in place to use real health data in training to support preparation for clinical practice.

## Introduction

### Background

Electronic health records (EHRs) are replacing paper medical notes in mature health care systems across the world [1]. EHRs are used in almost all interactions with patients in everyday clinical practice. Poor use of EHRs is associated with clinician burnout and adverse clinical consequences, highlighting the importance of high-quality EHR education to prepare health care professionals for the digital clinical environment [2,3].

Traditionally, clinical skills have remained the focus of undergraduate health care education [4]. Students are taught to take a history and examine and formulate differential diagnoses without considering the wealth of information present in an EHR [4]. In clinical practice, clinicians use EHRs as a central part of clinical decision-making to find patient information and gather data prior to seeing a patient [4]. The skills needed to do this should be taught in undergraduate curricula, and competencies have been published related to safe and effective EHR use [5,6]. However, health care students scarcely receive structured teaching to develop EHR-related skills; instead, they have variable exposure to electronic records during clinical rotations [7-11].

Where teaching is provided in classroom-based settings during undergraduate health care courses, fictional or “dummy” EHRs are often created for use [12]. These fictional records are reported to lack the complexity of real patient data and the depth of a real EHR. Whereas real EHRs capture the multidisciplinary nature of documentation, errors and coding this may be absent in fictional records, raising the possibility that real patient records could be of greater educational value in classroom-based teaching settings. The evolving role of EHRs, with the introduction of open notes in the United Kingdom meaning that patients can read clinician entries, adds complexity to EHR teaching [13,14]. Students need to be prepared for this change and become confident in working with the complexity of real patient data in this context. The use of real EHRs for teaching may however raise ethical and practical questions that need consideration.

### Objectives

In this paper, we explore what health care professionals, regularly using EHRs as part of their clinical work, think about the use of real EHRs for structured teaching purposes in classroom settings. Could the use of real records be a more valuable learning opportunity for undergraduate health care students in the United Kingdom?

Health Innovation Manchester is supporting our work across our 4 universities in the Greater Manchester region. Discussions were held with health care professionals to collect opinions regarding the use of real EHRs in delivering interprofessional EHR education to undergraduate health care students. These were used to gather participants’ own experiences of EHR education, their views on current EHR education, and the perceived benefits and challenges of using real EHRs in structured undergraduate teaching sessions. We present reflections from these discussions in this paper in order to inform and support the use of donated real patient records within undergraduate teaching.

## Methods

### Design

Two discussion sessions were hosted remotely via Microsoft Teams and audio-recorded. Both lasted for 90 minutes, beginning with a presentation from the moderator to explain the background and rationale for the proposal, followed by an hour-long facilitated discussion.

Participants were first contacted via word of mouth or through emails from colleagues who acted as gatekeepers. The only inclusion criterion was being a health care professional with a background in nursing, medicine, pharmacy, or an allied health role. The Participant Information Sheet and consent form were then emailed to individuals who expressed an interest in participating and met this criterion. Those who returned the signed consent form were invited to attend the discussions. In total, 10 participants were present across 2 multiprofessional discussion groups: 4 nurses, 3 doctors, 2 pharmacists, and 1 allied health professional (dietitian).

After considering availability and convenience for participants, the first group comprised 2 doctors, a nurse, and a dietitian, while the remaining participants were included in the second group. In total, 9 participants expressed the experience of supervising undergraduates or held formal teaching roles in health care courses. The moderator was a doctor with extensive experience in using EHRs in clinical practice; for the duration of her Academic Clinical Fellowship, she further studied literature on EHR education and collaborated with academics internally and across institutions to develop and improve teaching on EHRs for undergraduates. To gain a broad understanding of participants’ views, the moderator asked 3 open questions to prompt a discussion: (1) What are your thoughts on using real EHRs in education? (2) What are the benefits? (3) What are the drawbacks? The moderator listened to the audio recordings and summarized the points raised in this paper (Multimedia Appendix 1). Please note, this is not research; hence, a formal qualitative analysis of the discussions was not undertaken. Generative artificial intelligence was not used to write this paper. The GRIPP2 (Guidance for Reporting Involvement of Patients and the Public 2) checklist for reporting Patient and Public Involvement and Engagement was used to present reflections from the roundtable discussions (Checklist 1).

### Ethical Considerations

After reviewing the University of Manchester’s ethics requirements and further consulting with colleagues from the research ethics department, it was confirmed that this project did not require ethical review, as it fell under the remit of “Working With Professionals” rather than a research activity. However, as advised by the ethics department, all participants were provided with Participant Information Sheets and returned signed consent forms in order to partake in the discussions and provide permission for the use of direct quotes. Participation was voluntary and unpaid.

## Results

### Participants’ Past Experiences and Current Practices in EHR Education

Participants shared personal experiences of learning to use EHRs when they were students and discussed recent trends in EHR teaching as undergraduate tutors.

#### Past Experiences in EHR Education

Participants had no or limited experience of EHRs during their undergraduate training. Participant 10 reported using paper notes when he was completing medical training. He gained skills in EHR use in a self-directed and unstructured fashion, never having received guidance or education:

... there’s no structure to it. However, there is probably a little bit of on the job guidance of right, this is where you find this ...

[Participant 10, general practitioner]

Others shared experiences of gaining skills in the use of live EHR systems through experiential learning on clinical placements. Participant 9, for instance, recalled a helpful simulation session as a medical student in which he used an EHR system:

I was thinking back to when I did my medical degree, which was quite a while ago now ... we paired up and they had to see what it was like to try and do a 10-minute consultation where you were simultaneously typing and coding stuff. So, we all got, you know like (a) test login. So, I did find that a useful experience ... So I think we don’t teach them enough, but I had some positive experiences as well.

[Participant 9, general practitioner]

#### Current Practices in EHR Education Described by Participants

Participants reported that students gained exposure to EHRs through a variety of resources and settings, with some receiving logins for live EHR systems on clinical placements. Participants also pointed out that fellow clinicians or IT staff were often tasked to show students how to navigate EHRs, most often through a basic orientation and using fictional records. Participants felt that this was useful to introduce students to systems without overloading them. Some limitations were also highlighted:

... but obviously it’s not useful at all in terms of clinical ... so yeah, I can understand how that’s quite limiting in terms of not being able to get any kind of clinical information, experience, exposure from a dummy patient.

[Participant 2, pharmacist]

A distinction was made between “software training” consisting of a brief tour of an EHR system and teaching higher-level skills associated with EHR use such as generating high-quality data and critiquing documentation [4,5]. Participants felt that fictional records were not deemed to be useful for clinical learning or for developing higher-level skills required for the competent use of EHRs. Areas not addressed in the current EHR teaching were highlighted and included teaching on how to document and code. A delay in record access was also voiced; students failed to receive logins at the beginning of their placements.

A nurse participant described teaching her students using screenshots of real records. However, she highlighted the constraints of this approach; students could not view peripheral EHR systems, which contain information about other parts of the patient journey, for example, letters accessible on a different platform.

### Challenges in EHR Education

#### Absence of National Standards

Participants made recurrent observations that health care students are not taught how to document or how to interpret documentation. They felt that part of the challenge was the lack of national guidance on this. Additionally, individual clinicians document in different ways, with further interprofessional differences in writing styles.

#### Changing Function of Medical Records

Participants identified the evolving function of EHRs as another barrier to teaching. Earlier functions were to document clinical care to follow a patient’s journey and for medicolegal reasons. However, the purpose of EHRs has since transformed to allow patients to view and engage with their records, but this adapting role of EHRs is not taught to students.

... about two weeks ago in primary care, the function of medical records changed completely because all patients have access to them. So effectively someone overnight has changed the function of medical records instantly.

[Participant 3, general practitioner]

Participants commented that with the introduction of open notes, patients can now access and read free-text entries in their records. They felt that currently this created ambiguity on how best to document. Participant 3 commented that he was now writing out “essays” in the record to ensure its readability to patients. Participants highlighted differences in patient access to EHRs across the National Health Service (NHS); for example, patients may not be able to see their notes in secondary care.

#### Diversity in EHR Systems

Participants frequently cited diversity in EHR systems as a barrier to education; students may end up learning to navigate a system but not using it in clinical practice. One participant felt that unless there is a unified EHR provider across the NHS, EHR teaching would be futile. She shared her own disorienting experience of using different systems on clinical rotations.

... but with the software systems, it was almost like the first day of school every time I moved ... we need to have parity across the patch and using, you know either one or two or if not the same system.

[Participant 7, nurse]

One participant responded that the current policy is to let markets decide on EHR providers in the NHS, and a unified system creates greater vulnerability to cyberattacks, adding that accelerations in technology mean that EHR systems constantly change. Participants then agreed that teaching should focus on transferable skills, applied across EHR systems.

So, I’ve used Emis for about 8 or 9 years and I’ve recently gone to SystemOne, using that regularly. So, the way I assess for a patient is still transferable. Yes, obviously finding things is different and you know all the nitty gritty and the way it looks and stuff like that. But yeah, so the key point is more the skills of using it and what you get out of it ...

[Participant 10, general practitioner]

All participants acknowledged the value of learning fundamental EHR skills such as documenting in records, accessing data, and using EHRs to communicate within the multidisciplinary team (MDT). This would not replace software training that introduces students to a particular EHR system at the beginning of a placement. Developing key EHR skills would help students to know what they could find, and software training would show them where to retrieve it.

#### Interprofessional Differences

EHR teaching can be delivered to interprofessional cohorts to simulate the workplace and develop interprofessional competencies. Participants recognized the challenge of delivering teaching to an interprofessional student cohort due to different documentation styles; participants commented that advanced nurse practitioners document differently to doctors, as an example. Participants suggested that the delivery of unified teaching would require agreement between professions on core EHR documentation skills. It was felt that if a basic standard on documentation was taught, all students would understand patient notes regardless of their role. One participant explained how EHRs can facilitate interprofessional learning, communication, and understanding of different professional roles.

... I think that one of the biggest opportunities that is missed whenever a student comes to a placement, [...] trying to be part of MDT or even in an informal way, what came out in the ward round this morning, what’s the status of the patient’s discharge? As a dietitian, I would not be aware of those things when I was on my placements. Even though I could hear dietitians talking about it, I wouldn’t have really known how to find that piece of information and why it was important to me ...

[Participant 8, dietitian]

Overall, participants felt teaching should address the differing requirements of specific professions. However, there should also be opportunities for interprofessional EHR learning to prepare students for real clinical practice.

### Important Considerations for EHR Education

#### Minimum Standards

One participant commented that EHR education should establish a minimum standard of teaching, with additional clinical exposure to EHRs as a bonus:

... so we need to make sure that we are teaching a minimum level and then everything that happens in the (hospital) bases or on placement elsewhere then becomes that extra bit. So [...] I think what we really want to be looking at is to try and find what that minimum standard is and see if we can define that to teach that as a baseline.

[Participant 4, pharmacist]

He noted that other aspects of clinical learning such as the way we assess students are universal, but that exposure to EHRs depends on clinical placements, an area that needs addressing for parity.

#### Clinical Context

The facilitator suggested that initial EHR teaching would use a handful of real records, acknowledging that different clinical settings produce different records; for example, learning from primary care records would be different to that from secondary care records. Participants were asked for their views on using a small number of real primary care records. Participants agreed that holistic EHR education should reflect the varying clinical contexts of records. They noted that primary care records do contain some data from secondary care such as discharge summaries or bloods. One participant mentioned that it would be helpful to include these in teaching to give students a complete perspective of the patient journey.

#### Integration Into Existing Curricula

Participants advocated for the integration of EHRs into existing health care curricula as the most powerful to build student value of EHR teaching, avoiding repetition and duplication of teaching.

### Using Real Patient Records in EHR Education

#### Learning Opportunities From Using Real Patient Records in EHR Education

Participants highlighted multiple learning opportunities when using real patient records in undergraduate teaching. One participant identified the opportunity to learn from the longitudinal care of patients when using real records; students can view a patient’s journey in a short time. Another participant noted the benefits of learning from real-world time frames, appreciating processing times for results and addressing urgent versus routine tasks. Similarly, another participant noted that learning from real records can encourage students to critique data and learn about mistakes:

I don’t know if you do much learning in medical school from significant events, (be)cause I think that’s quite a useful way to learn about what can go wrong and why it goes wrong, which you know you can look at it from the point of view of records. You can look at cases where things have gone wrong because of the records.

[Participant 3, general practitioner]

Facilitators also elaborated on the value of learning from real errors; teaching could explore the potential clinical consequences of mistakes within EHRs. However, they suggested that there is often too much emphasis on things going wrong, which causes scaremongering and negativity. Instead, students could be aware of the potential for harm while appreciating that the record is a beneficial tool intended to aid clinicians.

Participants highlighted that another benefit of using authentic EHRs in teaching was learning from the complexity within the records. One nurse illustrated the invaluable opportunity of learning about holistic patient care and the real-life complexities of medicine through student interaction and learning from the complexities within real electronic systems. Conversely, another participant (pharmacist) commented that there may be “missing” information in real records, which can hinder learning. A facilitator responded that although this is not ideal for teaching, it may prepare students for real-world clinical practice where letters or data within a system may be incomplete.

Finally, a participant highlighted that entries in real records can demonstrate how the MDT communicates collaboratively to facilitate patient care:

…the multidisciplinary aspect of how everybody has integrated into that … care and what different people are doing and where they might fit into that care as well is really important. And then the communication aspect as well…. in terms of how you write up the record and how you communicate that either to a patient or things like referral notes, all of that stuff that gets put onto the system.

[Participant 4, pharmacist]

#### Concerns Surrounding the Use of Real Patient Records in EHR Education

Participants expressed their concerns about using real records in EHR education. This included exposing students to the entirety of authentic patient records at once, which could be overwhelming. Facilitators responded that this reflects the nature of real clinical practice. However, facilitators agreed that EHR learning should be progressive like learning clinical skills.

Participants also held concerns about potential litigation or reputational damage for clinicians:

… as an accountable practitioner, there’s always that fear of litigation. So, the idea of, perhaps my work, which I know that I’ve done to the best of my knowledge, and I’ve done as safely as possible. The idea of that being scrutinised by several people and I perhaps cannot explain what’s written in that manner there and then, that does not personally sit comfortably with me because you’re almost not able to explain your rationale for doing so.

[Participant 7, nurse]

Participant 10 (general practitioner) commented that documentation may be brief due to time pressures or human factors, and on these occasions, clinical entries are unrepresentative of usual practice. To this, another general practitioner remarked that students can already read clinical entries on placements; thus, using entries in didactic teaching is not vastly different. However, he did concede that it may expose clinicians to critique from larger student groups. A senior lecturer and pharmacist also explained that there are opportunities for medical students at his institution to raise concerns about clinician entries or other matters in a professional manner.

Despite the earlier-mentioned concerns, some participants held no objections to their names appearing on EHRs for teaching, viewing it as an opportunity to gain constructive feedback. A participant noted that this chimed with existing processes, where clinicians may access past entries by colleagues when a patient transitions between general practitioner surgeries.

Facilitators emphasized the importance of deidentifying EHRs to safeguard patient confidentiality. Participants raised concerns about the potential discovery of significant incidental findings in deidentified records, posing ethical dilemmas if patients cannot be identified. Many participants advocated for the tracing of entries back to patients to address errors in their records, emphasizing a duty of care toward patients. However, a facilitator explained that those entries in records often present nuanced situations rather than clear-cut scenarios. Without direct involvement in patient care, interpreting whether harm occurred due to reading record entries was deemed subjective. The facilitator cautioned against overreacting to perceived errors, suggesting that acting on such errors could lead to false alarms, as most patients are managed safely. Participant 3 expressed disagreement with this perspective.

So, I think most things won’t be black and white, but I think there are cases where it is black and white, and you probably won’t find them in the records. But I think I could, I could give you a case scenario where if I told you it, you would want to be able to de-anonymize the notes. I could- you know give me 5 minutes and I’ll generate one for you because I think you know there are issues that could occur where you- if you found it, you would feel liable to inform someone.

[Participant 3, general practitioner]

Participant 3 felt that certain events were highly likely to recur, with significant potential consequences. He recalled an anecdote of a missed blood result in a pregnant female that led to a negative outcome. Many participants expressed that this exemplified a powerful argument for deanonymizing EHRs if potential errors were found.

One participant suggested discussing the possibility of incidental findings with patients. She suggested that patients donating their records for teaching should be informed that if potential errors be found within records, these would not be enacted upon due to the aforementioned limitations.

Finally, participants expressed concerns about unethical practices by students such as inappropriate retrieval or use of real data. Facilitators responded that safeguards would be implemented to prevent this, but as in real life, there may be missed instances. Ultimately, participants concluded that we are preparing students for exactly this scenario—to handle real data! They also suggested that students should receive teaching on professional standards when handling digital information such as those outlined by the Health and Care Professions Council [15].

## Discussion

### Principal Findings

We are collaborating with Health Innovation Manchester and 3 other higher education institutions to create and implement a framework for a donation of real patient records for undergraduate education. These roundtable discussions with health care professionals brought important issues to the forefront to guide this process. We will use salient points raised in these reflections to inform data governance processes and anticipate and address potential benefits, risks, and roadblocks to consider when using real patient records for undergraduate education.

Whereas currently, real EHRs are not used for teaching in classroom environments, the benefits of using authentic records were discussed in detail. Participants explained that by using real records, students can learn about clinical decision-making and the causes and consequences of mistakes made in real contexts. Furthermore, students will learn to cope with the imperfection and “noise” within real EHRs; this includes the variety and complexity of data within authentic records but also its inaccuracies. Real EHRs also demonstrate team dynamics and multiprofessional communication through entries and contributions from colleagues. Challenges of using real EHRs in education were highlighted, including overwhelming students with vast volumes of data. By far, the main point of contention was the ethical and practical implications of incidental findings within records. Arguments for deidentifying records and informing patients in the case of clinically significant errors were presented, and contrarily, there were discussions about the subjective nature of entries and the potential for false alarms. Overall, it was suggested that in either scenario, patient donors should be informed of the process for handling incidental findings at the time of consent. Another challenge of using real records was compromising the privacy of clinicians; there were concerns about the risk of reputational damage and the potential for litigation.

It is clear that EHRs have an expanding audience in health care systems across the world [1]. The Regenstrief Institute in the United States previously incorporated a repository of pseudonymized authentic patient records for use by higher education institutes to teach clinical and digital competencies. To the best of our knowledge, a similar resource is not available for didactic teaching in the United Kingdom. In the United Kingdom, primary care data may be used for health care research on the premise of presumed consent, but the same principle has not been explored regarding the use of data in education [16].

Patients in the United Kingdom have recently gained default access to EHRs. This has caused considerable anxiety among professionals who have expressed uncertainty on how to document for both patients and clinicians and was corroborated by a participant in our discussion [17]. Clearly, the changing purposes and audiences of EHRs require new skills and nuanced teaching on documentation; health professionals may not be best placed to provide this. We as health care educators propose that honest discussions and reflections with students on open notes are important while central guidance is awaited.

Our discussion group with health care professionals revealed areas of agreement and areas of tension and concern among participants regarding the use of real data for EHR education. The principal area of ethical concern and debate was whether a process of deidentification of patient records should be undertaken when errors are found within records. The duty of candor outlines that patients should be informed of medical errors, but this is fraught with complexity in this particular educational context.

Participants voiced fears of scrutiny of individual entries, which could lead to litigation or reputational damage for clinicians. Participants cited the lack of opportunity to explain the context of their judgment when records are read and interpreted by people not directly involved in the patient’s care. However, it was noted that students already have access to EHRs on placements; hence, this is no different to accessing EHRs in classroom teaching, albeit the latter may involve detailed scrutiny of individual entries. Despite this concern, not all participants felt that clinician identities should be protected, as this would prevent an opportunity for constructive feedback on their practice. Realistically, if a large repository of EHRs is created, it would be impractical for us to provide feedback to all clinicians who have contributed to the records, and this would distract from the purpose of the resource.

Determining the threshold at which records would be deidentified is one problem, aside from the practicalities and logistics involved. Having clear processes and a consensus on dealing with these issues early on is paramount. It is imperative to obtain explicit consent from patients, and permissions from clinicians, and data handlers for accessing and using the data, including addressing the issue of incidental findings. Ensuring that students are adequately trained and familiar with the professional standards for handling real data is another key component.

Participants also highlighted the benefits of learning from mistakes made in real records, an opportunity that is lacking in synthetic records used for teaching. Identifying and managing errors is an important skill for future health care workers. Participants emphasized that synthetic records are simplistic compared to real EHRs, as real records confer the advantage of revealing the intricacies of health care and can demonstrate the complexity and volume of data encountered in real clinical practice.

Participants expressed that real records were complex and difficult to navigate, but interestingly, this was viewed as an advantage. Having the ability to navigate through the various components of a real record, appreciating the wealth of information available and the disconnect in communication, across health care settings was deemed as a powerful learning tool. Having a “snapshot” of a record, whether real or synthetic, lacks this benefit. Furthermore, students can learn about time frames and prioritization from the time intervals captured in real records. Finally, real records allow easy access to the multitude of entries by different professions, allowing students to learn from and work within an MDT. Overall, there was consensus that authentic EHRs are a better resource to prepare students for real clinical practice.

Our participants voiced a recurrent concern that EHR teaching lost value due to variability in EHR platforms used across health care systems. However, participants distinguished providing “software training” that teaches students to use specific EHR platforms from EHR education, focusing on underlying digital principles and transferable skills [4,5]. Participants highlighted that different clinical settings use diverse EHRs; educational goals and EHRs need to align for useful and transferable clinical teaching. Having access to records from a variety of settings would allow students to revisit different points of a patient’s journey and understand the holistic care delivered by the MDT.

Integrating EHRs into current curricula could create further opportunities to develop clinical reasoning and critical thinking skills, for example, by critique of trends such as blood test results over time. It could also encourage students to perceive its value in clinical work as well as avoid duplication in teaching. EHRs may also be used in examinations and assessments; if used in this way, EHRs should not appear as a tick-box exercise, as this risks undermining the value of learning EHR skills.

Interprofessional teaching is paramount to emulate real-world experiences and practice. Unsurprisingly, participants expressed the challenging nature of interprofessional teaching on EHRs. Each professional cohort uses EHRs differently, and consequently, there is variation in the data entered, documentation styles, as well as the nature of information accessed and relevant to each clinical role. Participants recognized the need for an agreement on basic EHR principles and the benefits this brings to an MDT. Students need to learn to use the EHR as a key tool for communication across disciplines.

Participants agreed that the absence of national guidance on documentation in EHRs makes it difficult to teach this important skill. Personal writing styles mean that the same encounter can be captured variably, making it difficult to define an individual style of documentation as “best practice.” While health care professionals rely on protocols for other aspects of clinical work, there is an absence of such a blueprint for documentation. Standard principles of documentation traditionally taught, such as ensuring legibility, dating, and signing handwritten entries, are also redundant in EHRs.

Participants alluded to coding during our discussions. Coding via Systematized Nomenclature of Medicine Clinical Terms is a crucial component of EHRs. It ensures that interoperability and its practical implementation have been expanding, helping to introduce clinical decision support systems over recent years [18-20]. Systematized Nomenclature of Medicine coding has been highlighted as a tool for improving the quality of data entered in electronic records [18]. Although further research is needed on its effect on patient outcomes, it is a core component of digital health systems, and a basic understanding of its principles is needed by clinicians. To our knowledge, there has been no reported assessment of Systematized Nomenclature of Medicine Clinical Terms coverage in undergraduate curricula in recent years; in view of our discussions with health care professionals, we anticipate health care curricula to be deficient in this aspect, and this area of EHR practice was not discussed in detail by participants taking part in our discussion.

### Limitations

We captured a variety of opinions that have helped outline the benefits and concerns around the use of real EHRs in classroom-based teaching. However, our findings represent the views from a small number of health care professionals within the United Kingdom, and professionals elsewhere may have differing views and experiences. Our work was confined to the collection of the views of health care professionals, and future work could explore the views of students and patients on how EHR teaching may be delivered.

### Conclusions

Modern clinical practice demands digital competencies alongside traditional clinical skills. Our work with health care professionals provides new insights into the potential role of real patient records in modern health care education. Unsurprisingly, participants expressed limited exposure to formal EHR teaching during their own undergraduate training, in part due to the relatively recent digitization of the NHS. Discussion of current educational trends revealed a variety of opportunities for undergraduates to develop EHR-related skills including exposure to EHRs on placements, teaching using screenshots of real records, and use of dummy records for IT-related software training. The exposure to the entirety of authentic patient records is acknowledged as reflective of real-life practice. Concerns that real EHRs may contain incidental findings, which are interpreted as harmful or leading to error, may be mitigated by informing data donors that incidental findings will not be acted upon, by teaching students professional standards for handling digital information, and by recognizing that EHR data are an interpretation of care but do not capture every occurrence or nuance in clinical encounters.

Overall, students will need a strong clinical foundation before learning to extract and use the information presented to them in real EHRs. Hence, EHR skills should be taught in a step-wise manner as with other clinical teaching. Real records can be used flexibly or adapted in a similar way to synthetic records for teaching; for example, in the early years, students may benefit from looking at 1 aspect of the record, whereas more experienced students may be exposed to a range of entries and expected to interpret these accordingly. If used appropriately and tailored to the learning needs of students, there is no reason for the complexities within real records to hinder teaching.

This [the record] *is* the practice of medicine. It’s intertwined with it. It determines what you do in the long run. You’re a victim of it or you’re a triumph because of it. The human mind simply cannot carry all the information about all the patients in a practice without error. And so the record becomes part of your practice.

[Larry Weed]

The above quote by Larry Weed, creator of the Subjective, Objective, Assessment and Plan style of documentation, emphasizes the crucial influence of EHRs on clinical practice. It is vital that our students are taught the skills required for effective EHR use in an age where the creation and manipulation of digital health data are ever-increasing. Using authentic data to teach health care students will bring education abreast of clinical practice. Our reflections on discussions with health care professionals outline important considerations for the responsible and effective use of real data in undergraduate teaching. We hope that this will provide a foundation for a framework that confronts and mitigates the issues that may arise when using authentic data for education, especially in the context of data presented within EHRs.

## Supplementary material

10.2196/60789 Multimedia Appendix 1 Supplementary data field containing raw field notes of roundtable discussions.

10.2196/60789 Checklist 1 GRIPP2 (Guidance for Reporting Involvement of Patients and the Public 2) reporting checklist.

## Abbreviations

EHR: electronic health record
GRIPP2: Guidance for Reporting Involvement of Patients and the Public 2
MDT: multidisciplinary team
NHS: National Health Service

## References

[1] Slawomirski L, Lindner L, Kd B, Haywood P, Hashiguchi TCO, Steentjes M (2023). Developments in OECD Countries as of 2021 OECD Health Working Papers.

[2] Muhiyaddin R, Elfadl A, Mohamed E (2022). Electronic health records and physician burnout: a scoping review. Stud Health Technol Inform.

[3] Dixit RA, Boxley CL, Samuel S, Mohan V, Ratwani RM, Gold JA (2023). Electronic health record use issues and diagnostic error: a scoping review and framework. J Patient Saf.

[4] McMillan B, Davidge G, Nadeem F, Dowding D, Wilson K, Davies A (2023). Navigating the electronic health record in university education: helping health care professionals of the future prepare for 21st century practice. BMJ Health Care Inform.

[5] Pontefract SK, Wilson K (2019). Using electronic patient records: defining learning outcomes for undergraduate education. BMC Med Educ.

[6] Biagioli FE, Elliot DL, Palmer RT (2017). The electronic health record objective structured clinical examination: assessing student competency in patient interactions while using the electronic health record. Acad Med.

[7] Gibson CM, Kwon HI, Tatachar A (2019). Impact of a low-cost simulated electronic medical record on perceptions of APPE readiness. Curr Pharm Teach Learn.

[8] Cook K, Cochran G, Gali H, Hatch T, Awdishu L, Lander L (2021). Pharmacy students’ readiness to use the electronic health record: a tale of two institutions. Curr Pharm Teach Learn.

[9] Vlashyn OO, Adeoye-Olatunde OA, Illingworth Plake KS, Woodyard JL, Weber ZA, Russ-Jara AL (2020). Pharmacy students’ perspectives on the initial implementation of a teaching electronic medical record: results from a mixed-methods assessment. BMC Med Educ.

[10] Elliott K, Marks-Maran D, Bach R (2018). Teaching student nurses how to use electronic patient records through simulation: a case study. Nurse Educ Pract.

[11] Ives AL, Tucker SR, Trovato JA (2020). Using electronic health record technology to teach inpatient medication order verification to pharmacy students. Am J Pharm Educ.

[12] Samadbeik M, Fatehi F, Braunstein M (2020). Education and training on electronic medical records (EMRs) for health care professionals and students: a scoping review. Int J Med Inform.

[13] Blease C, McMillan B, Salmi L, Davidge G, Delbanco T (2022). Adapting to transparent medical records: international experience with “open notes”. BMJ.

[14] Vanka A, Johnston KT, Delbanco T (2025). Guidelines for patient-centered documentation in the era of open notes: qualitative study. JMIR Med Educ.

[15] (2024). Standards of conduct, performance and ethics. Health and Care Professions Council.

[16] Riordan F, Papoutsi C, Reed JE, Marston C, Bell D, Majeed A (2015). Patient and public attitudes towards informed consent models and levels of awareness of electronic health records in the UK. Int J Med Inform.

[17] Blease CR, Kharko A, Dong Z (2024). Experiences and opinions of general practitioners with patient online record access: an online survey in England. BMJ Open.

[18] Gaudet-Blavignac C, Foufi V, Bjelogrlic M, Lovis C (2021). Use of the systematized nomenclature of medicine clinical terms (SNOMED CT) for processing free text in health care: systematic scoping review. J Med Internet Res.

[19] Sutton RT, Pincock D, Baumgart DC, Sadowski DC, Fedorak RN, Kroeker KI (2020). An overview of clinical decision support systems: benefits, risks, and strategies for success. NPJ Digit Med.

[20] Al-Hablani B (2017). The use of automated SNOMED CT clinical coding in clinical decision support systems for preventive care. Perspect Health Inf Manag.

