# Supplementary material for: Using Real Electronic Health Records in Undergraduate Education: Roundtable Discussion
Source: JMIR Form Res. 2025 Jun 12;9:e60789. doi: 10.2196/60789 (PMC12178567; doi:10.2196/60789)
Supplement: Multimedia Appendix 1 [file formative-v9-e60789-s001.docx]

**Using real patient records in EHR education: field notes**

1. **Current and past trends in EHR education**

Participants shared personal experiences of learning to use EHRs when they were students. They also discussed recent trends in EHR teaching as tutors for undergraduate students.

Past trends

While EHRs were not widely used when some participants were undergraduates, others had limited exposure to EHRs as students. One participant recalled a helpful session in which he used an EHR system in a simulated consultation to practice documenting while seeing patients for 10 minute appointments. Others used live EHR systems on clinical placements or developed EHR skills on the job; for example, one participant developed a structure for assessing patients using the record but was not taught this as a student.

Current trends

Current undergraduate students are commonly given logins for live EHR systems on clinical placements. Other clinicians or IT staff usually show them how to navigate the system. Some felt that these orientation sessions are fairly rudimentary and there is no teaching on higher level skills; students also do not learn about fundamental concepts such as Snowmed codes. Also, students may not receive logins until several weeks into their placements, highlighting the lack of parity with clinical learning.

One doctor described running parallel surgeries with students in General Practice. His students learn a lot about patients from their records before even seeing them. A nurse participant teaches her students using screenshots of real records. However, she explained the constraints of this approach as they cannot view peripheral EHR systems which contain information about other parts of the patient journey eg Docman.

As well as accessing live records, some students are given a basic orientation to EHR systems using fictional records (eg. Mickey Mouse). It was felt that this is especially useful to introduce them to systems without overloading them. However, this was not useful for clinical learning or for developing higher level skills for using EHRs.

1. **Challenges in EHR education**

Absence of national standards

A recurrent observation was that healthcare students are not taught how to document or how to interpret documentation. Part of the challenge is that there is no national guidance on how to document. Additionally, individual clinicians document in different ways and there also are interprofessional differences in writing styles. Also, with patients now an audience of the record, one participant mentioned that one of three options is possible: writing records using layman’s language, automatic abbreviation converters built into EHR systems or employing AI converters which create a translated version for patients. Until there is clarity on this, patients having access to their notes creates further ambiguity on how to document.

Diversity in EHR systems

The vast diversity in EHR systems was frequently mentioned as a barrier for EHR education. It was initially felt that students may end up learning a system that they will never need in clinical practice. One participant felt that unless there is a unified EHR provider employed across the NHS, EHR teaching would be futile. Similarly, accelerations in technology mean that EHR systems will constantly change. However, the ground reality are multiple, scattered EHR systems and there are no imminent plans to change this. Therefore, teaching should focus on transferable skills that can be applied to all EHR systems. Furthermore, it is unlikely that the NHS will use a single system as current policy is to let markets decide on EHR providers and a unified system creates greater vulnerability to cyber attacks. Additionally, there is significant overlap in basic elements of EHRs e.g. the type of data contained and their purpose. Hence, there is value in teaching the key [published competencies](https://pubmed.ncbi.nlm.nih.gov/30670000/) for using EHRs.

At the end of the discussion, all participants understood the logic of learning fundamental EHR skills. It was also mentioned that ‘software training’ would still take place at the beginning of placements to introduce students to EHR systems they haven’t used before. However, if they have developed key EHR skills, they will know ‘what’ they are trying to find and software training will simply show them ‘where’ to look for it. To illustrate this, a participant shared his experience of switching from Emis to TPP – he explained that the way he assesses a record is still transferable.

Current plans are to use TPP to deliver EHR teaching. Participants discussed if students would be familiar with this system. TPP is used by approximately 50% of GP surgeries and hence there is benefit in choosing an EHR provider that is used in real clinical practice for authenticity. However, it is unlikely that all students would be familiar with it due to variability in exposure to EHRs in undergraduate training. Many participants advised that a demo of TPP would be needed before teaching begins. From their experiences of teaching, if students are not familiar with a system, this can block their learning and cause anxiety. Similarly, another participant shared anecdotes of students and clinicians struggling to use HIVE when it was first adopted across their Trust. They are now considering giving students an orientation on HIVE before placements begin.

Changing function of medical records

Another barrier to EHR teaching is the evolving function of EHRs. The earlier functions were to document clinical care, to follow a patient’s journey and for medicolegal reasons. However, the purpose of EHRs has since adapted. As an example, it was mentioned that accelerated records’ access mean that patients can read entries. Consequently, a GP is now writing out ‘essays’ in the record, reflecting a change in the way that the record is used. Also, records are used differently across the NHS; for example, patients may not be able to see their notes in secondary care.

Currently, the adapting role of EHRs is not taught to students. However, any teaching on EHRs would need to incorporate changes in how the record is used.

Interprofessional differences

It is challenging to deliver teaching to an interprofessional student cohort as requirements can vary considerably. It was interesting to note that some EHR systems seem far simpler than others. Furthermore, there are differences in documentation styles e.g. ANPs document differently to doctors and there are also differences within specialties. To deliver unified teaching on this, there needs to be agreement between professions on basic requirements. It was felt that if a basic standard on documentation is taught, then all students will understand patient notes, regardless of their role. Furthermore, real clinical practice demands a good understanding of different professional roles and recognising differences in the way colleagues work. Interprofessional EHR teaching would provide students an opportunity to learn about the roles of their colleagues and the multidisciplinary approach to patient care. A participant shared that as a student dietitian, she didn’t understand communication from other professions. Interprofessional EHR teaching allows an opportunity to teach communication with colleagues.

Overall, it seems that teaching should address the requirements of specific professions as these differ. However, there should also be opportunities for interprofessional EHR learning to prepare students for real clinical practice.

1. **Important considerations for EHR education**

Minimum standards

EHR education should establish a minimum standard of teaching. It was noted that the way we assess students is universal, but exposure to records is not and depends on clinical placements. As with other aspects of clinical learning, students should receive a certain minimum level of teaching on electronic records for parity so that any additional clinical exposure to EHRs is a bonus.

Clinical context

Different clinical settings produce different records, eg learning from primary care records is very different to records from ICU or secondary care. EHR education should reflect the varying clinical contexts of records for holistic teaching. It was explained that eventually, the aspiration is to use EHRs from different clinical settings but initial stages would involve teaching with a handful of records only. Also, participants mentioned that primary care records do contain some data from secondary care e.g. discharge summaries / bloods. It would be helpful to include these in teaching to give students a complete perspective of the patient journey, for example absence of bloods from secondary care may imply that a patient has a new anaemia when in fact they don’t – this is also a good learning point!

Integration into existing curricula

Participants advised integration of EHRs into existing healthcare curricula. This has significant benefits. Firstly, it would help students to perceive the value of EHR teaching. Students are still in ‘student mode’ and care about exams and defined competencies eg medical licensing agreement. Also, integration into current curricula would avoid repetition and duplication of teaching.

1. **Using real patient records in EHR education**

Learning opportunities with real records

- Opportunity to learn from mistakes made in records – there is no structured teaching on this currently. Aim to do this without disparaging colleagues. Students should be aware of things that go wrong but this should be framed as a positive learning experience, rather than further adding to paranoia.
- Students can learn from longitudinal care of patients -with the benefit of hindsight, they can cover a patient’s journey over years in a short space of time.
- Learning about realistic timeframes eg. how long do bloods take to come back, when to escalate if secondary care hasn’t responded, what is urgent.
- Multidisciplinary learning- how the MDT works for patient care. Students can appreciate everyone’s role and where they can contribute to a patient’s journey through entries and contributions of colleagues to the record.
- Communication – how to communicate with patients and with colleagues.
- Students can learn from the real-life complexity of records– ‘chaotic’ nature of notes and complex patient stories such as polypharmacy.
- Students can learn about the ‘chaos’ introduced by systems - for example having to use or document in two systems that aren’t connected.
- Allows a wider and more accurate perspective on the patient’s journey – eg nursing student was shown spirometry results but when read in historical medical context it was interpreted differently.
- Provide an opportunity for self-directed learning, evidence shows this is better than traditional learning where students are handed all the information– students can manipulate the record themselves.
- Authenticity – what happens in real practice, for example ‘missing letters’.
- Students will learn to interrogate and question records appropriately, for example a patient may be coded as having a diagnosis which was self-made.

Concerns surrounding use of real records

- Too complex and overwhelming, however this is real clinical practice! EHR learning should be progressive like learning clinical skills, there should be a gradual build-up in of skills rather than exposure to whole records at once.
- Fear of litigation – clinicians are not present to explain their management or clinical care. Their management may be interpreted incorrectly, also documentation may be brief due to the reality of working in the NHS - human factors and time pressures.
- Clinicians may be afraid that their reputation will be tarnished if tutors criticise their work infront of students. Students can already read entries however, this will be on a much larger scale and currently, entries not being ‘analysed’.
- There is a safe space for students to raise issues with entries in a professional way, for example clinical debrief sessions. Students taught to have these conversations in an appropriate way.
- Some clinicians would not mind having their names on records as it may give them an opportunity to receive feedback on their work for self-improvement. Not different to what is already happening -when a patient moves surgeries, clinicians at new surgery can see all past entries.
- Incidental findings in records– one of the reasons we don’t do ECGs on eachother or take bloods from other students is because of the possibility of incidental findings. This raises ethical issues, many participants felt that the patient should be informed if they are at risk of harm – duty of care towards patient and professional responsibility.
- This can also be used as an opportunity for students to learn – if an error was made, what would you do with that now?
- One participant has a rare condition – the research team can access her records but she had to agree that if something was picked up incidentally, they would de-anonymise her record so they can inform her. Does the opposite need to be in place?
- Most entries in records are not ‘black and white’ but ‘grey’. Difficult to know if someone will or has come to harm by reading an entry that can be interpreted differently.
- Certain events have a high likelihood of recurrence with high potential consequence. These are black and white – an example given of a missed diagnosis of hyperthyroidism that possibly led to a stillbirth in a patient.
- Tutors should be competent to teach using real records – some may not feel confident with this.
- Cannot access real patient data without permission – need permission from data handler. Example in one participant’s workplace, it was flagged up when colleagues accessed notes out of hours for case studies.
- Limitations of real systems eg in Emis you can’t see the role of the person documenting – difficult to interpret entries if this is the case. Also if you don’t have access to all the supporting systems like Docman it can be confusing to understand the clinical information. However, even if that can’t be achieved, may be useful for students to think about ‘missing data’ and what to do in these cases.
- Unethical practice by students – students may capture or use data inappropriately. Should be safeguards in place for this, but ultimately there may be students who slip through the net. Important to teach students about ground rules and professionalism. Ultimately, we are preparing students to handle real data! HCPC guidance on professionalism.

Overall, there are some concerns around fear of litigation and damage to reputation of clinicians. It was suggested that as a minimum, clinician names should be removed from the record and participants were accepting of this. Learning from mistakes within records should be framed without disparaging colleagues and in a constructive manner that makes students aware of the limitations of real life clinical work.

There was a lot of discussion around the possibility of incidental findings and the ethical issues this raises. Participants were not comfortable with the idea of not informing patients of potential errors. Facilitators suggested that the purpose of EHR education is to teach and for students to learn from the patient’s journey. Raising errors within the record would detract from the purpose of this project. Furthermore, it is extremely difficult to appreciate what happened versus what is written in the record – difficult to judge whether a patient will come to harm without having ever seen the patient or without speaking to clinicians looking after the patient – potential for many false alarms. Also, in reality any possible ‘errors’ are subtle rather than catastrophic. It was suggested that we may need medicolegal advise for this. Ultimately, there needs to be a clear pathway and discussion around dealing with this potential occurrence.
