# Supplementary material for: Using Real Electronic Health Records in Undergraduate Education: Roundtable Discussion
Source: JMIR Form Res. 2025 Jun 12;9:e60789. doi: 10.2196/60789 (PMC12178567; doi:10.2196/60789)
Supplement: Checklist 1 [file formative-v9-e60789-s002.docx]

GRIPP2 short form

| Section and topic | Item | Reported on page No |
| --- | --- | --- |
| 1: Aim | Report the aim of PPIE in the study | 3 |
| 2: Methods | Provide a clear description of the methods used for PPIE in the study | 3 |
| 3: Study results | Outcomes—Report the results of PPIE in the study, including both positive and negative outcomes | 4 |
| 4: Discussion and conclusions | Outcomes—Comment on the extent to which PPIE influenced the study overall. Describe positive and negative effects | 10 |
| 5: Reflections/critical perspective | Comment critically on the study, reflecting on the things that went well and those that did not, so others can learn from this experience | 12 |
